# Urine-to-Blood Partitioning of Per- and Polyfluoroalkyl Substances in Human Biomonitoring: Implications for Environmental Exposure Analysis and Bioaccumulation Assessment

**DOI:** 10.3390/molecules31111880

**Published:** 2026-05-30

**Authors:** Peiyao Ye, Hexiang Bai, Jing Shi, Zhaomin Dong, Kai Luo

**Affiliations:** 1 Key Laboratory of Environmental Medical Engineering, Ministry of Education, School of Public Health, Southeast University, Nanjing 210009, China; 220234066@seu.edu.cn (P.Y.); 230268611@seu.edu.cn (H.B.); dongzm@seu.edu.cn (Z.D.); 2 Suzhou Center for Disease Control and Prevention, Suzhou 215000, China; lhjyk@szcdc.cn

**Keywords:** per- and polyfluoroalkyl substances (PFAS), blood-urine ratio, protein binding affinity, bioaccumulation, environmental health

## Abstract

Per- and polyfluoroalkyl substances (PFAS) are persistent chemicals with substantial bioaccumulation potential, but their distribution between blood and urine in humans remains poorly characterized. In this review, we assessed the urine-to-blood concentration ratio (UtBCR) as a potential indicator of PFAS bioaccumulation by integrating evidence from human biomonitoring studies and protein-binding data. We summarized PFAS concentrations in human serum and urine across general and highly exposed populations and identified clear compound-specific differences in blood–urine partitioning. We further examined the associations of UtBCR with carbon chain length, biological half-life, and binding-related parameters for human serum albumin (HSA), liver fatty acid-binding protein (L-FABP), and several renal transporters. Pairwise correlation analysis and partial least squares regression indicated that UtBCR was closely associated with major toxicokinetic determinants, particularly protein-binding affinity, carbon chain length, and biological half-life. Parameters related to FABP, HSA, urate transporter 1 (URAT1), and organic anion transporter 4 (OAT4) showed more consistent associations with UtBCR than those related to organic anion transporters 1(OAT1) and organic anion transporter 3 (OAT3), suggesting that plasma/tissue binding and tubular reabsorption may contribute more than active tubular secretion to PFAS blood–urine partitioning. Overall, UtBCR appears to be a useful toxicokinetic metric for comparing the relative bioaccumulation potential of PFAS.

## 1. Introduction

In recent decades, per- and polyfluoroalkyl substances (PFAS) have been widely used in various industrial and consumer products—such as food packaging, stain-resistant textiles, firefighting foams, and lubricants—due to their exceptional chemical stability, hydrophobicity, and thermal resistance. Their highly persistent molecular structures render them resistant to environmental degradation, leading to their designation as “forever chemicals” [1,2]. According to the Organization for Economic Co-operation and Development (OECD), over 4700 PFAS compounds had been identified globally by 2017 [3]. With the advancement of high-resolution mass spectrometry (HRMS) and non-targeted analytical techniques, an increasing number of PFAS compounds have been identified. The United States Environmental Protection Agency (U.S. EPA) CompTox Chemicals Dashboard listed 14,735 PFAS compounds with explicit chemical structures (as of August 2022) and 1915 PFAS chemicals without explicit structures (as of March 2024) [4,5]. Meanwhile, an increasing diversity of PFAS species has been widely detected in human populations, thereby raising significant public health concerns [6,7,8,9,10].

Throughout this review, we use the terms “legacy PFAS” and “Emerging PFAS” as follows. Legacy PFAS refers to long-chain perfluoroalkyl acids—principally perfluoroalkyl carboxylic acids with ≥7 perfluorinated carbons (e.g., PFOA, PFNA, PFDA) and perfluoroalkane sulfonic acids with ≥6 perfluorinated carbons (e.g., PFHxS, PFOS)—that were widely produced and used from the 1950s through the early 2000s and are now subject to international restrictions under the Stockholm Convention and equivalent regulations. Emerging PFAS or PFAS alternatives refer to compounds introduced more recently as replacements for legacy PFAS, including: (i) short-chain homologues (perfluoroalkyl acids with ≤6 perfluorinated carbons for PFCAs or ≤5 for PFSAs, e.g., PFBA, PFBS, PFHxA), (ii) perfluoroalkyl ether acids and sulfonates (e.g., HFPO-DA (GenX), HFPO-TA, ADONA, 6:2 Cl-PFESA (F-53B), cC6O4, PFMOAA), and (iii) ultra-short-chain PFAS such as TFA (C2) (Table S1). This terminology follows the framework adopted by the OECD (2018) [3] and authoritative reviews [1,11,12]. It should be noted that the legacy/emerging distinction is operational rather than strictly chemical: it reflects production history and regulatory status, and a given compound may be classified differently across jurisdictions and time periods.

One of the most critical environmental health characteristics of PFAS is their bioaccumulative potential. Epidemiological studies have demonstrated that certain PFAS, such as perfluorooctanoic acid (PFOA, C8) and perfluorooctane sulfonate (PFOS, C8), exhibit prolonged biological half-lives in humans—often spanning several years—and tend to accumulate in the blood, liver, and placenta [10,13,14,15,16]. Due to their widespread exposure and limited excretion, PFAS bioaccumulation has become a major focus in environmental health research. An increasing number of studies have provided evidence linking PFAS exposure to a range of adverse health outcomes, including liver and kidney dysfunction [17,18,19,20], immune suppression [21,22], metabolic and endocrine-disrupting impacts [23,24], reproductive toxicity [25], cardiovascular disease [26,27], and increased cancer risk [28,29,30,31], especially among populations with high exposure levels [32].

The bioaccumulation of PFAS in the human body is influenced by multiple factors, with plasma protein binding affinity recognized as a primary determinant [33,34]. Experimental studies have demonstrated that PFAS can bind to human serum albumin (HSA) and fatty acid binding proteins (FABPs), and that the binding constant—or its inverse, the dissociation constant (Kd)—is correlated with their retention time in the body [35,36]. By binding to these proteins, PFAS reduce their free plasma fraction, thereby decreasing glomerular filtration and urinary excretion while increasing systemic retention [37,38]. Long-chain PFAS (e.g., those containing eight or more carbon atoms) typically exhibit stronger protein-binding affinities and longer biological half-lives—a trend consistently observed in both animal and human studies [39,40,41,42].

In biomonitoring efforts, PFAS exposure in human populations is typically evaluated using blood/serum and urine samples. Blood/serum PFAS concentrations are generally considered indicators of total body burden, whereas urinary concentrations reflect excretion rates [43,44,45]. The urine-to-blood concentration ratio (UtBCR) has been suggested as a toxicokinetic parameter, representing the balance between accumulation and elimination, and analogous urine-to-serum ratios have previously been employed to characterize the renal excretion of caffeine [46] and fluoride [47]. However, whether this ratio can serve as a reliable surrogate marker for PFAS bioaccumulation remains uncertain, particularly given the variability in protein-binding affinities and carbon chain lengths among PFAS compounds.

To address this knowledge gap, the present study integrates data from the literature across multiple PFAS compounds to explore the relationships between urine-to-blood concentration ratios and key physicochemical and toxicokinetic parameters, including carbon chain length, binding affinities to HSA and FABP, and biological half-life. Throughout this review, we use the notation Cn to denote the number of perfluorinated carbon atoms in each PFAS molecule (e.g., C4 for PFBA, C8 for PFOA and PFOS), to facilitate cross-compound comparison of chain-length–dependent toxicokinetic behavior. The aim is to assess the scientific validity of using UtBCR as an empirical toxicokinetic indicator of PFAS urine–blood partitioning and urinary elimination tendency, and to explore its relationship with compound-specific properties relevant to persistence and bioaccumulation, thereby contributing complementary information for exposure assessment and health risk characterization.

## 2. Profiles of Commonly Detected PFASs in Human Serum

Widespread biomonitoring initiatives worldwide have consistently detected PFAS in human serum, highlighting both their environmental persistence and the extent of human exposure. Serum concentrations of PFAS are widely regarded as reliable biomarkers of internal exposure, owing to their strong binding affinity for plasma proteins and prolonged biological half-lives.

### 2.1. Global Baseline: The Legacy Quartet (PFOS, PFOA, PFHxS, PFNA)

In the United States, the National Health and Nutrition Examination Survey (NHANES) has tracked serum PFAS concentrations on a national scale since 1999 [48]. The data of NHANES indicate that PFOS (C8), PFOA (C8), PFHxS (C6), and PFNA (C9) are the most commonly detected congeners, with detection frequencies consistently exceeding 95% in every monitoring cycle and geometric mean levels typically in the nanograms per milliliter (ng/mL) range among the general population.

Comparable biomonitoring programs in Canada, Australia, and Europe, such as the Canadian Health Measures Survey (CHMS), the Australian Human Biomonitoring programs (Australian HBM), the HBM4EU project, the German Environmental Survey (GerES), and the Norwegian Mother, Father and Child Cohort Study (MoBa), have also reported high detection frequencies of PFAS in blood across populations, with PFOS, PFOA, PFNA, and PFHxS typically representing the major contributors to overall PFAS burdens [49,50,51,52,53,54]. For example, in HBM4EU-aligned studies (2014–2021), serum or plasma samples from 1957 adolescents (12–18 years) across nine European countries showed detection frequencies above 60% for each of these four compounds [55].

Nationally representative biomonitoring from the China National Human Biomonitoring Program (CNHBM) indicates that, in 2017–2018, PFOA and PFOS were the dominant PFAS in Chinese blood, with median concentrations markedly higher than those of other congeners. The next most prominent compounds included 6:2 Cl-PFESA (F-53B, C8), PFNA, PFDA (C10), and PFHxS, generally at approximately ng/mL levels [56,57,58,59]. Notably, 6:2 Cl-PFESA, a substance widely used in China’s electroplating industry, shows substantially higher detection frequency and concentration in Chinese populations than in U.S. and European populations. In the 2017–2018 NHANES cycle, 6:2 Cl-PFESA (F-53B) was detected in only 7.98% of U.S. participants (with >44% of detections self-identifying as Asian), whereas CNHBM data suggest a ~100% detection rate in China [48,60,61].

### 2.2. Global Consistency and Regional Heterogeneity of PFAS Exposure

Large-scale human biomonitoring across regions worldwide consistently identified the same “legacy quartet”—PFOS, PFOA, PFHxS, and PFNA—as the dominant serum PFAS, supporting the concept of a global baseline of legacy PFAS exposure. However, beyond this shared legacy baseline, the PFAS mixture shows clear regional fingerprints, likely driven by differences in industrial structure, chemical substitution pathways, and regulatory timelines. A prominent example is 6:2 Cl-PFESA (F-53B) mentioned above. In serum samples from a non-occupationally exposed general population in Shandong Province—one of China’s most developed regions for the fluorochemical industry—13 PFAS (PFOS, PFOA, PFHpA (C7), PFNA, PFDA, PFUnDA (C11), PFDoDA (C12), PFTriDA (C13), PFHxS, HFPO-TA (C9), 4:2 Cl-PFESA (C6), 6:2 Cl-PFESA, and 8:2 Cl-PFESA (C10)) were detected in >97.9% of participants. Notably, the emerging alternatives HFPO-TA and 4:2/6:2/8:2 Cl-PFESA showed significantly higher detection frequencies and concentrations than the national averages reported by population-based biomonitoring [62]. Therefore, PFAS exposure patterns in human blood exhibit a “dual” feature: legacy PFAS show global consistency (a ubiquitous background exposure), whereas alternative PFAS display pronounced regional heterogeneity (regional fingerprints).

### 2.3. PFAS Substitution Transition and Trends in Blood Exposure Profiles

As production and use of legacy long-chain PFAS (e.g., PFOS, PFOA, and some long-chain PFCAs/PFSAs) have been progressively restricted, industry has increasingly adopted a range of emerging alternatives, including short-chain homologues and fluoroether PFAS (e.g., ether-containing replacement acids/sulfonates), to maintain performance in applications such as surface treatments, flame retardancy, stain resistance, and processing aids [1,63,64]. This substitution-driven transition is reshaping human blood PFAS profiles: while concentrations of several legacy long-chain compounds have declined in some regions, certain short-chain and ether PFAS are being detected more frequently in blood, and their relative contributions are increasing in specific populations and regions—consistent with systematic shifts in exposure sources and chemical-use patterns. In the U.S. NHANES, over roughly two decades (from 1999–2000 to 2017–March 2020), the serum geometric mean concentrations of the same “legacy quartet” (PFOS, PFOA, PFHxS, and PFNA) declined by 87%, 74%, 52%, and 16%, respectively. However, NHANES first reported population serum concentrations of five short-/mid-chain or ether PFAS—PFHpS (C7), PFHxA (C6), HFPO-DA (GenX, C6), ADONA (C7), and 9CLPF (6:2 Cl-PFESA)—in 2017–2018 [48]. Although their detection frequencies and concentrations were lower, these findings nevertheless attracted substantial attention. Comparable declines in PFOS and PFOA have been reported in general-population and background-exposure studies from Canada, Germany, Denmark, and Australia, despite differences in study design, population composition, and the timing and stringency of regulation [53,54,65,66]. Trend magnitudes and shapes vary by region (e.g., earlier peaks, faster or slower declines, and compound-specific fluctuations). However, the cross-country consistency indicates that production phase-outs and use restrictions have lowered population-level body burdens of legacy PFAS. By contrast, evidence for offsetting increases in short-chain homologues and ether-based PFAS alternatives in blood is less consistent and more difficult to compare across countries because monitoring programs differ over time in analyte coverage and detection limits.

Across studies of Chinese serum PFAS in the past decades, legacy compounds remain dominant, but profiles are shifting. A national synthesis (1996–2022) indicates PFOA increased over time (*p* = 0.087), whereas PFOS showed a weak decline (*p* = 0.83), alongside rising PFAS alternatives (e.g., 6:2 Cl-PFESA, *p* = 0.36; 8:2 Cl-PFESA, *p* = 0.24) [67]. Longitudinal data from Hubei (Dongfeng–Tongji cohort) confirm compositional change from 2008 to 2018: median PFOA doubled from 0.81 to 1.83 ng/mL, while linear PFOS decreased from 5.38 to 4.32 ng/mL; 6:2 Cl-PFESA remained stable (~1.44 ng/mL). Short-chain PFCAs became more prevalent, as reflected by higher detection frequencies: PFBA rose from 51.4% (2008) to 64.8% (2018), and PFHpA from 17.6% to 34.7% [43].

### 2.4. Characterization of Short-Chain and Emerging PFAS by Blood Biomonitoring and Associated Challenges

Although the studies summarized above show declining blood concentrations of legacy long-chain PFAS—typified by PFOS and PFOA—these compounds still account for the largest share of PFAS in blood. At the same time, short-chain PFAS and emerging PFAS alternatives contribute relatively little to the overall PFAS burden. This does not imply low real-world burdens of short-chain or emerging replacement PFAS. Instead, differences in bioaccumulation and biological half-life determine how effectively blood biomonitoring captures individual PFAS. These toxicokinetic differences—together with legacy background exposure driven by environmental persistence and heterogeneity in analyte coverage across monitoring programs—can cause short-chain and replacement compounds to appear less prominent in blood. In this context, trifluoroacetic acid (TFA, C2), an ultra-short-chain PFAS, has attracted increasing scientific attention [68]. A small human biomonitoring study (*n* = 81) in Indiana, USA, reported that TFA was the predominant perfluoroalkyl acid (PFAA) in serum samples (detection frequency, 74%; median concentration, 6.0 ng/mL) and accounted for 57% of the total serum PFAA concentration [45]. Although TFA is a ubiquitous environmental contaminant, interpreting serum TFA concentrations is challenging, particularly for source attribution. Unlike many legacy PFAS, whose serum concentrations often reflect direct exposure, serum TFA may be partly derived from endogenous formation from multiple precursors. Potential sources include biotransformation and degradation of longer-chain PFAS side chains and other fluorinated compounds (e.g., the widely used inhalational anesthetic isoflurane) [69,70], which complicates separation of direct environmental exposure from metabolic production.

Table 1 summarizes the studies cited in Section 2.1, Section 2.2, Section 2.3 and Section 2.4 and integrates large-scale or representative biomonitoring evidence on legacy and emerging PFAS in major countries and regions worldwide, including their detection patterns and concentration trends.

### 2.5. Sex and Age Differences in Serum PFAS Concentrations

Human biomonitoring studies across regions also report sex- and age-specific differences in serum PFAS concentrations. Men generally have higher levels than women, most consistently for legacy compounds such as PFOS, PFOA, PFNA, and PFHxS [54,57,67,71,72,73,74,75,76,77,78,79]. This pattern is often attributed to female-specific elimination and transfer pathways—menstruation, pregnancy and childbirth (placental transfer), and lactation—that reduce circulating concentrations [80,81,82,83,84]. Sex differences in toxicokinetics (e.g., renal elimination), as well as occupational and behavioral exposures, may also contribute [85]. With respect to age, biomonitoring studies generally indicate a clear age gradient in serum PFAS concentrations, although the direction and magnitude are compound- and period-specific. For legacy long-chain PFAS (e.g., PFOS and PFOA), cross-sectional data from different countries commonly show increasing concentrations with age or higher levels in middle-aged and older adults [48,54,78,86,87,88]. This pattern is typically attributed to their long biological half-lives (on the order of years) and higher environmental inputs in earlier decades due to more widespread production and use, which together produce marked “cohort effects” and long-term accumulation. In some older adults, reduced renal function and cessation of menstruation may further reduce PFAS elimination, thereby increasing circulating levels [81,89,90]. In contrast, some short-chain PFAS and emerging alternatives do not necessarily exhibit the same monotonic age-related increase in blood; instead, age differences may be weaker or show more variable patterns, potentially reflecting shorter residence times in the body, exposures driven more by contemporary local sources (e.g., impacted water supplies, specific consumer products, or indoor dust) [45], and limited comparability due to recent changes in target analytes and detection limits. Overall, age-related differences can be viewed as the combined result of “toxicokinetics (half-life, distribution, and elimination) × historical and contemporary exposure × biomonitoring methodology”. Notably, in the blood of Chinese populations, 6:2 Cl-PFESA shows a clear sex difference—higher levels in men than in women, and a significant age-related increase [57,60,91]. This finding indicates that 6:2 Cl-PFESA exhibits toxicokinetic properties in humans that are highly similar to those of legacy long-chain PFAS (e.g., PFOS), suggesting that it is not a “safer, more readily degradable” alternative.

**Figure 1:**
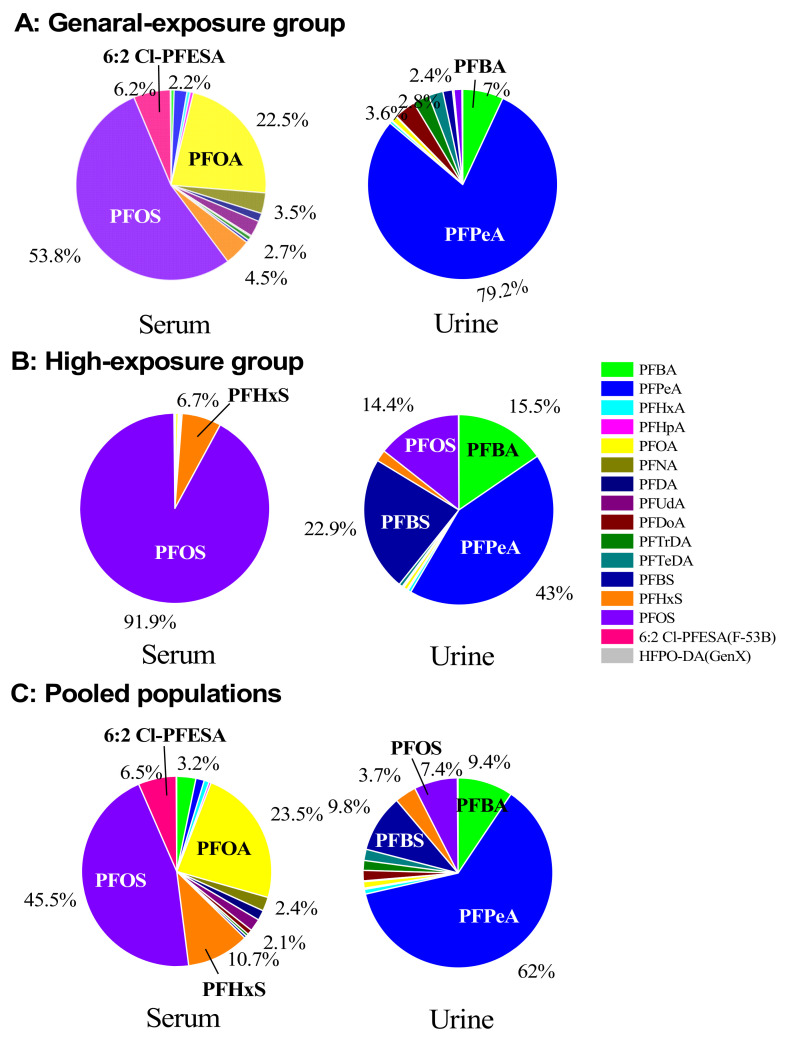
Relative composition profiles of PFAS in human serum (**left panels**) and human urine (**right panels**) under general-exposure, high-exposure, and pooled-population scenarios. The distributions were constructed based on the mean concentrations reported in the paired urine and blood biomonitoring studies. When mean concentrations were not available, median concentrations were used instead.

The relative composition of PFAS in human serum under general-exposure, high-exposure, and pooled-population scenarios is shown in Figure 1A–C (left panels); see Section 3 for the corresponding urinary profiles. Table 2 summarizes the concentration ranges of PFAS in human serum reported across selected biomonitoring studies [39,40,42,44,45,85,91,92,93,94,95,96,97,98,99,100,101,102,103,104,105,106,107,108,109,110,111,112,113,114,115,116,117,118,119,120,121,122,123,124,125,126,127,128,129,130,131,132,133,134,135,136,137,138,139,140,141].

## 3. Profiles of Commonly Detected PFASs in Human Urine

While blood (serum) remains the gold standard for assessing body burden of legacy long-chain PFAS due to their high protein binding affinity and extended half-lives, it often fails to capture the rapid turnover of short-chain homologues and emerging ether-based alternatives. Consequently, although urinary biomonitoring data for PFAS remain significantly less abundant than those for blood/serum, urine has emerged as a critical, complementary matrix for characterizing the “shifting” PFAS internal exposure. The toxicokinetic partitioning between blood and urine is governed by renal clearance mechanisms; specifically, the efficiency of resorption from the glomerular filtrate via organic anion transporters (OATs) [142,143,144,145]. Long-chain compounds, such as PFOS (C8) and PFOA (C8), exhibit high affinity for serum albumin and are efficiently reabsorbed by renal OATs, resulting in lower urinary excretion [145]. Conversely, short-chain PFAS and certain ether-based alternatives exhibit lower protein binding and faster renal elimination rates, making urine the preferred matrix for capturing these transient, recent exposures (Figure 1) [146].

Despite variations in study populations and exposure backgrounds across different epidemiological and biomonitoring studies, the PFAS profile in human urine predominantly exhibits a distinct “short-chain” characteristic compared to blood. This is primarily manifested in both detection rates and concentrations across two key aspects: In cross-matrix comparisons, short-chain PFAS (especially C4 PFBA, C5 PFPeA, and C6 PFHxA) demonstrate significantly higher detection rates and concentrations in urine than in blood. Conversely, legacy long-chain PFAS (such as C8 PFOA and PFOS, C9 PFNA, and C6 PFHxS) display the exact opposite trend, with higher detection rates and concentrations in blood than in urine [38,44,45,85,120,122,123,124,125,126,127,128,129,131,132,147,148,149]. Meanwhile, in within-matrix comparisons, the detection rates and concentrations of short-chain PFAS surpass those of legacy long-chain homologues within the urine matrix. In contrast, within the blood matrix, long-chain PFAS dominate over their short-chain counterparts in both detection rates and concentrations [45,100,120,122,123,126,127,129,131,148,149,150,151,152,153,154,155]. Based on data from the 2013–2014 US NHANES, it demonstrated that short-chain PFAS such as PFBA (C4, 13.3%) and PFHxA (C6, 22.6%) were the most frequently detected compounds in urine; this sharply contrasts with long-chain PFAS, which are universally detected in serum but have urinary detection frequencies below 0.1% [148]. A clinical biomonitoring study involving 227 patients with liver diseases from Guangzhou, China, demonstrated that the detection frequencies of PFBA (C4) and PFPeA (C5) were remarkably higher in urine (97% and 86%, respectively) compared to whole blood (8% and 36%, respectively) and serum (9% and 51%, respectively) [149]. The paired cord blood and neonate urine data (*n* = 80) further corroborate this pattern. Short-chain PFAS, namely PFBA (C4) and PFPeA (C5), were detected at substantially higher frequencies in urine (100% and 96.3%) than in cord blood (26.3% and 81.3%). Meanwhile, legacy long-chain PFAS like PFNA, PFHxS, and PFOS, despite being ubiquitous (100%) in cord blood, experienced a drastic decline in urinary detection frequencies to 73.8%, 25.0%, and non-detectable, respectively. Thus, short-chain PFAS overwhelmingly dominate the urinary matrix profile compared to their long-chain homologues [131].

While PFAS concentration profiles in occupational workers align with those of the general population—exhibiting short-chain dominance in urine and long-chain dominance in blood—the matrix-dependent discrepancy in detection frequencies is notably absent. Specifically, the detection frequencies of long-chain PFAS in urine and short-chain PFAS in blood increase markedly, with maximum rates exceeding 90% across both matrices [100,120,126,127,128,132,156]. For example, in He et al.’s (2023) [100] study of 72 fluorochemical workers, serum median concentrations of long-chain PFOA, PFOS, and PFHxS (194, 998, 1098 ng/mL) far exceeded short-chain PFBA, PFPeA (C5), PFHxA, and PFBS (C4) (3.92, 0.08, 1.14, 36.0 ng/mL). Conversely, in urine, these short-chain medians (12800, 2420, 7100, 8230 ng/L) were significantly higher than the long-chains (525, 238, 1500 ng/L). Meanwhile, detection frequencies for these PFAS across both matrices reached 93.1–100% [100]. This is primarily driven by the extreme exposure doses typical of occupational settings compared with those in the general population. Because high environmental exposure results in both long- and short-chain PFAS absolute concentrations in blood and urine consistently exceeding the analytical limit of detection (LOD), the simple presence-or-absence metric of detectability no longer effectively captures matrix-specific partitioning between urine and blood. Continuous occupational exposure compensates for the rapid renal clearance of short-chain homologues, sustaining their steady-state concentrations in serum well above the LOD. Theoretically, saturation of tubular reabsorption could also lead to increased urinary excretion of long-chain PFAS. A pharmacokinetic model developed by Andersen et al. (2006) [157] based on monkey data demonstrated that tubular reabsorption of long-chain PFAS is transporter-mediated and saturable. At low PFAS concentrations in the tubular filtrate, reabsorption is efficient and renal clearance remains minimal. However, as concentrations increase and approach the maximal transport capacity of these carriers, reabsorption becomes progressively saturated, leading to higher renal clearance [157]. By extension, occupationally exposed populations, who typically carry substantially higher internal PFAS burdens than the general population, are more likely to reach the saturable range of tubular reabsorption. This could increase the fraction of long-chain PFAS eliminated in urine and may partly explain the relatively high detection frequency of long-chain PFAS in urine from occupational cohorts. When Loccisano et al. (2011) [158] scaled this PBPK model to humans, incorporating the same saturable renal reabsorption mechanism with an increased transporter capacity (Tm), the model successfully reproduced measured serum PFOA concentrations across multiple exposed populations, confirming that this saturable process governs human PFAS pharmacokinetics [158]. However, this cross-species extrapolation from primate models to humans remains speculative. The elevated detection frequency of long-chain PFAS in human urine may also simply reflect a greater overall body burden rather than a true change in clearance efficiency. Distinguishing between these possibilities will require paired urine and blood data across populations with different exposure levels to determine whether the renal clearance fraction increases more than proportionally with exposure.

Urine is also an attractive matrix for biomonitoring ultra-short-chain PFAS such as trifluoroacetic acid (TFA, C2). In recent years, TFA has attracted increasing scientific and regulatory attention. Environmental concentrations of TFA have risen by more than an order of magnitude in the post-2010 period relative to pre-2010 levels [68], and several countries have begun to establish guidance values for TFA in drinking water, including 60 μg/L in Germany and 2.2 μg/L in the Netherlands [159,160]. In a study of pooled urine samples from Australia (*n* = 70 pools representing 6040 individuals), Muir et al. (2025) [139] used direct-injection ion chromatography with 13C2-TFA as the internal standard. They reported a 100% detection frequency, with a median concentration of 24 μg/L and a range of 3.4–300 μg/L [139]. In contrast, Zheng et al. (2023) detected TFA in only 31% of urine samples collected in Indiana, USA (*n* = 81), with a median concentration below the method detection limit (MDL) of 3.5 μg/L, although the concentration range (<3.5–290 μg/L) was comparable [45]. The marked difference in detection frequency is more likely attributable to analytical methodology than to true differences in population exposure. Zheng et al. (2023) [45] used weak anion-exchange solid-phase extraction (WAX) followed by ENVI-Carb cleanup, with 13C4-PFBA as a surrogate internal standard. Under these conditions, TFA recovery may be low and variable because TFA can be strongly retained by ENVI-Carb and is also susceptible to matrix effects from inorganic anions. By comparison, the dilute-and-shoot approach used by Muir et al. (2025) [139] is better at minimizing analyte loss and background interference during sample preparation. These urinary biomonitoring studies indicate that human exposure to TFA is widespread and urine is a suitable matrix for its biomonitoring. Meanwhile, robust analytical methods are essential for the accurate assessment of this ultrashort-chain PFAS [139].

It should be noted that TFA detected in urine does not necessarily originate exclusively from exogenous environmental exposure, but may also be formed in vivo through the metabolism of pharmaceuticals, inhalation anesthetics, and pesticides containing aryl-, heterocyclic-, or alkyl-CF3 groups [70,161,162,163,164]. For example, the widely used volatile anesthetic isoflurane and the antidepressant fluoxetine have both been shown to generate TFA through CYP-mediated biotransformation or microbial degradation [69,165]. In the Australian study by Muir et al. (2025) [139], older individuals (>45 years) exhibited significantly higher urinary TFA concentrations, together with greater inter-individual variability. Given the short urinary elimination half-life of TFA (16–42 h), the authors suggested that this elevation may be related to the more frequent use of C–CF3-containing medications in older adults, rather than simply reflecting increased environmental exposure [139]. Therefore, when urinary TFA is used as a biomarker of environmental exposure at the population level, the potential confounding contribution of drug metabolism should be carefully considered.

Another noteworthy group of urinary PFAS comprises emerging substitutes. In a Chinese neonatal cohort (*n* = 80), six alternative PFAS—6:2 Cl-PFESA (C8), HFPO-DA (C6), HFPO-TA (C9), PFMOAA (C3), PFO2HxA (C4), and PFO3OA (C5)—were detected in urine, with detection frequencies ranging from 16.3% to 100%, indicating that these emerging substitutes are already widespread in the environment and can enter the human body as early as the neonatal period. Distinct urinary-to-blood partitioning patterns further suggest substantial toxicokinetic heterogeneity among these compounds. For example, 6:2 Cl-PFESA (F-53B) was detected in 41.3% of neonatal urine samples, with a geometric mean concentration of 0.175 ng/L, markedly lower than in paired cord blood, where the detection frequency was 100%, and the geometric mean concentration was 588 ng/L, yielding a urine-to-blood ratio of 0.00029 [131]. A similarly low urinary partitioning was observed in fluorochemical workers (*n* = 72), in whom 6:2 Cl-PFESA was detected in 38.9% of urine samples at a mean concentration of 0.40 ng/L, compared with 100% detection and a mean serum concentration of 6760 ng/L in paired blood samples, corresponding to a urine-to-blood ratio of only 0.000059 [100]. These findings indicate extremely limited renal elimination and suggest that the bioaccumulative behavior of 6:2 Cl-PFESA resembles that of highly persistent long-chain PFAS such as PFOS. In contrast, perfluoro-2-methoxyacetic acid (PFMOAA), an emerging short-chain perfluoroalkyl ether carboxylic acid, showed much greater urinary partitioning. In neonates, PFMOAA was detected in 100% of urine samples, with a geometric mean concentration of 30.88 ng/L. In contrast, paired cord blood samples showed a detection frequency of 98.8% and a geometric mean concentration of 171.4 ng/L, resulting in a urine-to-blood ratio of 0.180 [131]. A similar pattern was observed for the ammonium salt of 2,2,3,3-tetrafluoro-2-(1,1,2,2,3,3-hexafluoro-3-(trifluoromethoxy)propoxy)acetic acid (cC6O4, C6). This compound represents a new generation of perfluoroalkyl surfactants and can serve as a substitute for traditional polymerization aids such as PFOA in fluoropolymer production. Among workers occupationally exposed during fluoropolymer manufacturing, cC6O4 was quantified in 100% of both urine and serum samples; median urinary concentrations ranged from 0.90 to 1.43 µg/L, whereas median serum concentrations ranged from 1.80 to 2.34 µg/L, giving urine-to-blood ratios as high as 0.39–0.79 [166]. Together, these results suggest that PFMOAA and cC6O4 are cleared more rapidly through urinary excretion and therefore exhibit substantially shorter biological persistence in humans. The pronounced differences in blood–urine partitioning among emerging substitutes indicate that these alternatives should not be regarded as inherently safer by default. Compound-specific toxicokinetic evaluations are needed to support substitution strategies and regulatory prioritization.

Biomonitoring studies of PFAS in human urine remain far less common than those based on serum, and long-chain PFAS are typically detected at low frequencies and concentrations in urine, making age- or sex-related differences more difficult to characterize consistently. Available evidence suggests that there is no uniform or robust pattern in the effects of sex and age on urinary PFAS levels across populations. Rather, the observed variability is more likely to reflect differences in recent exposure and urinary elimination kinetics than differences in internal body burden alone. Accordingly, urinary PFAS concentrations should be interpreted in the context of the compound class, individual physiological status, and, where possible, paired blood-based biomarkers, rather than being directly attributed to any single demographic factor.

Table 3 summarizes the distribution and concentrations of PFAS in the urine samples from the general population and high-exposure groups across various regions [39,40,42,45,85,100,120,121,122,123,124,125,126,127,128,129,130,131,132,136,137,138,139,151,152,167].

## 4. Linking the Urine-to-Blood Concentration Ratio to Protein Binding, Renal Transport, and Biological Half-Life

Synthesizing the human biomonitoring evidence reviewed above, PFAS exhibit clear compound-specific partitioning between blood and urine. Long-chain PFAS and some highly persistent emerging substitutes tend to be retained in the systemic circulation. In contrast, short-chain homologues and several emerging substitutes are more readily detected in urine. This cross-matrix contrast indicates that the relative distribution of PFAS between blood and urine reflects not only exposure patterns, but also the balance between systemic retention and renal elimination. Accordingly, compared with either matrix alone, the urine-to-blood concentration ratio (UtBCR) may provide a more informative toxicokinetic metric. Assessing this possibility requires moving beyond descriptive summaries of biomonitoring patterns to consider the mechanistic determinants of the ratio, particularly protein-binding affinity, interactions with renal transporters, and biological half-life.

**Figure 2:**
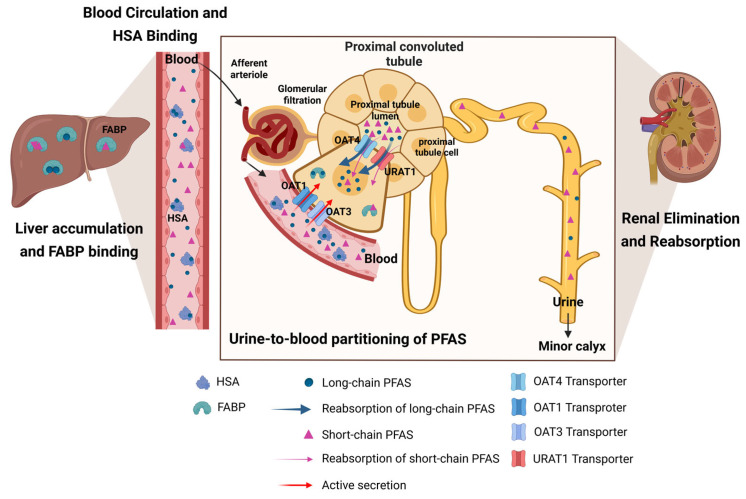
Schematic illustration of the toxicokinetic disposition of long- and short-chain PFAS, highlighting hepatic accumulation, protein binding, and renal tubular transport.

As the principal circulating binding protein, human serum albumin (HSA) can reduce the freely filterable fraction of PFAS, thereby promoting their persistence in blood, lowering the UtBCR, and increasing their bioaccumulation potential [168]. Fatty acid-binding proteins (FABPs), especially those expressed in the liver (L-FABP), may further promote intracellular retention and blood-dominant partitioning [36,169,170]. At the renal level, OAT1/OAT3-mediated active secretion may enhance urinary elimination, whereas OAT4- and URAT1-mediated reabsorption may reduce renal clearance and prolong body residence [144,171,172,173]. These processes provide a mechanistic basis for evaluating UtBCR as an indicator of PFAS bioaccumulation potential (Figure 2).

In this study, binding affinities to six proteins—hL-FABP, HSA, OAT1, OAT3, OAT4, and URAT1—and their associations with 16 PFAS (Table S2) UtBCRs were examined in the general-exposure group, the high-exposure group, and the pooled population. The UtBCR values were derived from paired blood–urine studies based on reported mean concentrations, with median concentrations used as substitutes when mean values were not available (Tables S3 and S4). Docking-derived binding energies were generated for 16 PFAS using AutoDock Vina 1.1.2 based on protein structures obtained from public databases (Text S1, Table S5), and experimentally measured dissociation constants for PFAS binding to HSA (Kd-HSA) and L-FABP (Kd-FABP) were compiled from the literature (Table 4, Text S2, Tables S6 and S7). Because UtBCR values spanned several orders of magnitude, log10-transformed UtBCR was used in subsequent analyses. PFAS half-life data were taken from the study by Abraham et al. (2024) [174]. We selected this source because its half-life estimates were based on direct human oral exposure experiments using 13C-labeled PFAS, and 15 PFAS were evaluated within a single experimental framework, providing better internal consistency and comparability than heterogeneous estimates pooled from multiple studies.

### 4.1. Pairwise Correlation Analysis

Pairwise correlation analysis showed that docking-derived binding energies for hL-FABP, HSA, OAT1, OAT3, OAT4, and URAT1 were generally positively correlated with UtBCR, with stronger correlations in the general-exposure and pooled populations than in the high-exposure group. In the general-exposure group, hL-FABP binding energy (BE-hL-FABP, r = 0.978), BE-URAT1 (r = 0.973), BE-OAT1 (r = 0.962), and BE-OAT3 (r = 0.960) showed particularly strong positive correlations. Kd-FABP was also consistently positively associated with UtBCR across all three populations (r = 0.802–0.807, all *p* < 0.01), whereas Kd-HSA showed weaker and less consistent associations. By contrast, PFAS chain length and biological half-life were negatively correlated with UtBCR, with the strongest association observed for chain length in the general-exposure group (r = -0.937, *p* < 0.01). These results suggest that stronger protein-related interactions are generally associated with lower UtBCR. In contrast, longer-chain and more persistent PFAS tend to exhibit lower urinary excretion relative to blood burden (Table 5). At the same time, the attenuation of correlations in the high-exposure group implies that PFAS urine–blood partitioning may be governed by more complex mechanisms under elevated exposure conditions.

These findings support the use of UtBCR as an informative toxicokinetic metric for characterizing PFAS urine–blood partitioning and relative renal excretion efficiency. However, UtBCR should not be regarded as a substitute for conventional bioaccumulation metrics such as BCF, BAF, KOW, or tissue–blood partition coefficients used in PBPK modeling. Instead, when interpreted together with compound-specific protein binding, transporter interactions, and persistence properties, UtBCR can provide complementary information for ranking PFAS according to their relative urinary elimination tendency and blood retention. In PBPK applications, observed UtBCR values may be used to calibrate or evaluate model-predicted urine-to-blood ratios and to constrain renal clearance-related parameters, including glomerular filtration, tubular secretion, and tubular reabsorption, rather than to directly define tissue partition coefficients.

### 4.2. Partial Least Squares Regression (PLSR) Analysis

**Figure 3:**
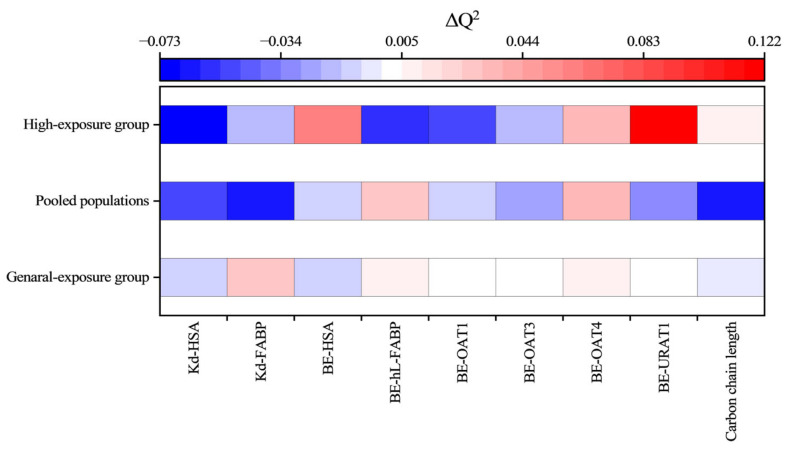
Contributions of protein-binding and transport-related variables to PLSR model performance for PFAS UtBCRs across exposure groups.

To account for collinearity among predictors and to evaluate their joint effects, we further applied PLSR analysis, using chain length, docking-derived binding energies, and the literature-derived Kd values as predictors and UtBCR as the response. The general-exposure model required only one latent component and showed strong explanatory and predictive performance (R^2^Y = 0.907, Q^2^ = 0.855), indicating that variation in UtBCR in this group could largely be explained by a relatively unified latent mechanism. Although variable importance in projection (VIP) values were uniformly low, leave-one-variable-out analysis showed that Kd-FABP contributed most to model prediction (ΔQ^2^ = 0.0247), followed by BE-OAT4 and BE-FABP, whereas BE-OAT1 and BE-OAT3 contributed little independently (Figure 3). This pattern suggests that FABP-related binding information, particularly experimentally derived affinity, may better capture PFAS partitioning in the general-exposure population than classical secretory transporters.

In contrast, the high-exposure model required four latent components and showed high goodness-of-fit but only moderate predictive ability (R^2^Y = 0.892, Q^2^ = 0.538), supporting a more complex, multi-mechanistic interpretation. Although VIP values were highest for BE-URAT1, Kd-FABP, carbon chain length, BE-HSA, and BE-OAT4, leave-one-variable-out analysis identified BE-URAT1 (ΔQ^2^ = 0.122), BE-HSA, and BE-OAT4 as the most stable positive contributors to prediction. BE-OAT1 and BE-OAT3 had negative ΔQ^2^ values, suggesting limited independent value and possible redundancy. Notably, Kd-FABP showed a high VIP (2.40) but a negative ΔQ^2^ (−0.171), indicating that it contributed to latent structure without improving cross-validated prediction.

The pooled model was intermediate, requiring three latent components and yielding the highest overall fit with good predictive ability (R^2^Y = 0.931, Q^2^ = 0.732). In this model, BE-OAT4 (ΔQ^2^ = 0.0367) and BE-FABP (ΔQ^2^ = 0.0273) were the most consistent contributors to prediction. In contrast, BE-HSA, Kd-HSA, BE-OAT1, BE-OAT3, and BE-URAT1 did not provide stable predictive gains despite relatively high VIP values for some of these variables.

Overall, the PLSR results indicate that the determinants of PFAS urine-to-blood partitioning differ by exposure scenario. UtBCR in the general-exposure group appears to be governed primarily by a relatively coherent latent mechanism, whereas the high-exposure group shows greater mechanistic complexity. Across models, OAT1 and OAT3 did not show stable independent contributions, suggesting that active tubular secretion may not be the dominant driver of inter-PFAS differences in UtBCR in this dataset. In contrast, FABP-, HSA-, URAT1-, and OAT4-related parameters showed more consistent relevance, supporting a greater role for plasma/tissue binding and tubular reabsorption-related processes in shaping PFAS urine–blood partitioning.

To evaluate whether the use of median values as substitutes for unavailable means influenced our findings, we performed a sensitivity analysis by re-compiling the dataset to include only studies reporting mean concentrations (Table S8 and Figure S1). Because all high-exposure studies originally provided mean values, this restriction affected only the general-exposure subset. Pairwise correlation analysis using the mean-only dataset yielded results that were essentially unchanged from the original analysis: the associations between UtBCR and protein Kd, molecular docking-derived binding energies, biological half-life, and carbon-chain length retained their direction, magnitude, and statistical significance, with the sole exception that the marginal correlation between general-population UtBCR and Kd–HSA shifted from *p* < 0.05 to non-significant. PLSR analysis on the mean-only dataset further demonstrated strong explanatory and predictive capability for general-population UtBCR (R^2^Y = 0.978, Q^2^ = 0.933), slightly outperforming the model that included median-based studies. Leave-one-variable-out analysis identified Kd–FABP as the dominant predictor (ΔQ^2^ = 0.0822), followed by the binding energies of FABP (ΔQ^2^ = 0.0345), HSA (ΔQ^2^ = 0.00831), and OAT3 (ΔQ^2^ = 0.000068), reinforcing the central role of PFAS–FABP interactions in shaping PFAS partitioning in the general population. Predictive performance for the pooled population declined modestly (R^2^Y = 0.898, Q^2^ = 0.528), likely reflecting binding-site saturation in high-exposure individuals, in whom elevated PFAS levels promote disproportionate accumulation in blood and weaken the coupling between UtBCR and binding parameters. Collectively, these results confirm that our conclusions are robust to the inclusion of median-based studies and that UtBCR is most informative as a toxicokinetic indicator in general-exposure populations.

## 5. Discussion and Conclusions

Pharmacokinetic studies in rats, mice, and non-human primates have shown that urinary PFAS concentrations are often higher than fecal concentrations, indicating that urine is an important excretion route [186,187,188]. Accordingly, the present study employs the UtBCR as the primary indicator of PFAS excretion. However, fecal excretion also represents an important elimination pathway, particularly for long-chain PFAS [174,189]. Recent evidence indicates that urinary and fecal excretion are compound-specific: renal clearance predominates for many short-chain PFAS and PFOA, whereas fecal elimination is more important for PFOS and long-chain PFCAs such as PFNA and PFDA. Thus, UtBCR may not fully capture total elimination when biliary/fecal clearance dominates [38,190].

In vitro studies, including molecular docking and equilibrium dialysis, have demonstrated that even short-half-life PFAS such as PFBS can bind to L-FABP and be taken up by hepatocytes [35,175]. In vivo evidence further indicates that hepatic PFAS concentrations correlate strongly with L-FABP binding affinity (R^2^ = 0.8 across 14 PFAS in mice) [191], and that the hepatic–blood distribution of PFAS in rats is governed by competitive binding to L-FABP and serum albumin, a finding considered extrapolatable to humans [192,193]. Our docking results show that the binding affinity of PFAS to HSA and L-FABP increases with carbon-chain length, supporting the preferential hepatic accumulation of long-chain PFAS. Once accumulated in the liver, long-chain PFAS can be excreted into bile and enter the intestine, where they may either be eliminated through feces or reabsorbed via enterohepatic circulation. Rapid biliary reabsorption of PFOA and PFOS has been experimentally confirmed and contributes substantially to their long biological half-lives [194,195].

Short-chain PFAS, in contrast, exhibit weaker protein binding, remain largely in the free fraction in blood, and are predominantly excreted via glomerular filtration [143]. Their reabsorption by renal transporters is also limited, as evidenced by their weaker docking affinities to URAT1 and OAT4 relative to long-chain PFAS, facilitating their elimination via urine. Collectively, both renal tubular reabsorption and enterohepatic circulation contribute to the bioaccumulation of long-chain PFAS, and future studies should integrate these two mechanisms to better elucidate PFAS internal disposition.

A key methodological limitation of the present analysis is the use of unadjusted (mass-based) urinary PFAS concentrations rather than creatinine- or specific-gravity-corrected values. This choice was dictated by the inconsistent reporting of correction factors across the source studies; nevertheless, it warrants critical evaluation. Variations in urinary dilution—driven by hydration status, sampling time, age, and renal function—can introduce non-trivial noise into urinary biomarker concentrations [196,197]. This noise is expected to be most pronounced for short-chain PFAS (e.g., PFBA, PFPeA, PFHxA, PFBS), which exhibit the highest UtBCR values and the most rapid renal clearance, and may therefore inflate the variance—and possibly the absolute magnitude—of their UtBCR estimates [39,198]. Importantly, urinary dilution is not expected to correlate systematically with PFAS chain length or protein-binding affinity, suggesting that the bias is primarily random rather than directional. Thus, the relative ranking of PFAS by UtBCR and the structural correlations between UtBCR and protein-binding parameters identified in our PLSR analysis should remain robust. However, the absolute UtBCR values—particularly for short-chain congeners—should be interpreted with appropriate caution. Moreover, because we pooled cohort-level mean concentrations rather than individual measurements, individual-level dilution variability is partially averaged out [199,200]. Future studies incorporating individual-level creatinine or specific gravity data would help refine UtBCR estimates and reduce residual measurement noise, particularly for rapidly excreted short-chain PFAS.”

A second important source of unmeasured variability in our meta-analyzed UtBCR estimates is inter-individual variation in renal function, which was not reported in most source studies. PFAS renal handling involves glomerular filtration of the unbound fraction, active tubular secretion, and tubular reabsorption mediated by renal transporters, including OATs, OAT4, URAT1, and OATP-related transporters [144,201]. Reduced GFR may decrease urinary PFAS excretion relative to serum concentrations by lowering the filtered load and prolonging systemic residence time. In contrast, selective tubular dysfunction may alter UtBCR in either direction depending on whether secretion or reabsorption is predominantly affected. Thus, the net direction of renal-function-related bias cannot be assumed a priori and may vary across PFAS congeners and clinical contexts. This issue is particularly relevant for high-exposure and older populations. In some high-exposure or susceptible populations, PFAS concentrations have been associated with altered kidney function or kidney injury biomarkers [202,203,204,205]; however, causal interpretation is complicated because impaired renal function may itself increase serum PFAS concentrations by reducing elimination. Therefore, the weaker UtBCR–binding parameter associations observed in high-exposure groups in this study may reflect not only protein-binding-site saturation, but also exposure-related or pre-existing differences in renal handling. Similarly, age-related declines in GFR and the higher prevalence of hypertension or diabetes among older adults may contribute to heterogeneity in UtBCR across general population cohorts with broad age ranges [206,207]. Because most source studies did not report estimated glomerular filtration rate (eGFR) or renal biomarkers, this source of variability could not be evaluated directly. Future toxicokinetic studies should report paired serum and urinary PFAS concentrations together with eGFR and, where possible, tubular injury biomarkers, to enable renal-function-stratified analyses and to disentangle protein-binding effects from renal-handling effects.

Taken together, UtBCR provides a practical, integrative indicator of renal excretion efficiency for PFAS. High UtBCR values, observed for short-chain PFAS, are associated with weak protein binding, limited tubular reabsorption, and short biological half-lives, indicating that these compounds are predominantly cleared via urine. In contrast, low UtBCR values, characteristic of long-chain PFAS, reflect strong protein binding, extensive tubular reabsorption, hepatic accumulation, and enterohepatic recirculation, which collectively underlie their long half-lives and bioaccumulation potential. UtBCR should therefore be viewed as a relative ranking metric of renal clearance efficiency rather than an absolute measure of half-life or biliary excretion; when combined with molecular docking against renal transporters and hepatic binding proteins, it offers a mechanistically informative framework for predicting PFAS internal disposition.

However, the gap between R^2^Y (0.892) and Q^2^ (0.538) in the high-exposure PLSR model indicates that this model is unsuitable for fitting the relationship between UtBCR and various transporters in this study, likely reflecting more than just mechanistic complexity. At the elevated serum PFAS concentrations characteristic of occupational and highly contaminated cohorts, both serum protein binding sites (albumin, FABPs) and renal tubular transporters (OAT1/OAT3, OAT4, URAT1, OATPs) are expected to approach saturation [144], introducing concentration-dependent nonlinearities that a linear PLSR projection cannot fully capture. Together with the smaller sample size and unmeasured heterogeneity in renal function discussed above, this likely explains the disproportionate reduction in Q^2^. Future studies with larger high-exposure datasets may benefit from nonlinear approaches, such as kernel-PLSR or mechanistic Michaelis–Menten models.

In conclusion, this study systematically assessed the urine-to-blood concentration ratio (UtBCR) of PFAS as a potential indicator of bioaccumulation by integrating human biomonitoring evidence with protein-binding data. UtBCR varied markedly across PFAS and was closely linked to key toxicokinetic characteristics, particularly protein-binding affinity, carbon-chain length, and biological half-life. Both pairwise correlation and PLSR analyses showed that parameters related to FABP, HSA, URAT1, and OAT4 were more consistently associated with UtBCR than those related to OAT1 and OAT3, suggesting that plasma/tissue binding and tubular reabsorption may play a greater role than active tubular secretion in determining PFAS partitioning between urine and blood in the present dataset. The general-exposure group displayed a relatively coherent underlying pattern, whereas the high-exposure group showed greater mechanistic complexity, indicating that the factors governing UtBCR may differ across exposure scenarios. Taken together, these findings support the use of UtBCR as an informative empirical toxicokinetic metric for characterizing PFAS urine–blood partitioning and relative urinary elimination efficiency. Rather than serving as a direct proxy for bioaccumulation potential, UtBCR should be interpreted as a complementary indicator that reflects the balance between blood burden and urinary elimination. When considered together with compound-specific protein binding, transporter interactions, and persistence properties, UtBCR may help inform the relative retention and elimination behavior of PFAS.

## 6. Limitations

Several limitations of this study should be noted. First, the analysis relied on biomonitoring and protein-binding data compiled from the literature rather than on individual-level paired measurements obtained under a unified study design, which may have introduced heterogeneity arising from differences in population characteristics, analytical methods, and exposure settings. Second, although renal transport and protein binding were considered, the potential impact of impaired kidney function on PFAS UtBCR was not directly evaluated. Renal dysfunction may affect glomerular filtration, tubular secretion, and tubular reabsorption, thereby altering PFAS partitioning between blood and urine and potentially influencing the observed associations. Third, to improve comparability across studies, urinary PFAS concentrations were analyzed as unadjusted mass concentrations rather than creatinine-corrected values. Consequently, UtBCR may be affected by urine dilution and differences in voided volume, which could contribute to additional measurement variability. Fourth, several mechanistic variables, including docking-derived binding energies, were estimated using in silico approaches and may not fully reflect in vivo interactions under physiological conditions. Finally, the number of PFAS included in some subgroup analyses was limited, which may have reduced statistical power and restricted the broader applicability of the findings. Further validation of UtBCR as an indicator of PFAS bioaccumulation potential will require standardized paired blood–urine datasets with detailed information on renal function, urine dilution markers, and individual toxicokinetic characteristics.

## Figures and Tables

**Table 1:** Regional patterns and temporal trends of legacy and emerging PFAS reported in human blood biomonitoring studies.

Country/Region	Reference	Study/Cohort Name	Legacy PFAS Detected	Emerging PFAS Detected	Temporal Rend
USA	[48]	NHANES	PFOA, PFOS, PFHxS, PFNA		PFOS, PFOA, PFHxS, and PFNA geometric mean concentrations decreased from 1999–2000 to March 2020
Indiana, USA	[45]	Human Biomonitoring Study		TFA	Detection rate = 74%, contributed 57% to the total serum PFAA concentration
Canada	[53]	CHMS	PFOA, PFOS, PFHxS, PFNA		PFOA and PFOS concentrations decreased
Australia	[54]	Australian HBM	PFOA, PFOS, PFHxS, PFNA		PFOA and PFOS concentrations decreased
Denmark	[65]				1990–2021: PFOA and PFOS concentrations decreased
Europe	[51]	HBM4EU	PFOA, PFOS, PFHxS, PFNA		
Germany	[49]	GerES	PFOA, PFOS, PFHxS, PFNA		
	[66]				1982–2010: PFOA and PFOS concentrations decreased
Norway	[50]	MoBa	PFOA, PFOS, PFHxS, PFNA		
China	[56,57,58,59]	CNHBM	PFOA, PFOS, PFNA, PFDA, PFHxS	6:2 Cl-PFESA	Concentrations: PFOA, PFOS > 6:2 Cl-PFESA > PFNA > PFDA > PFHxS
	[67]				1996–2022 concentrations: PFOA, 6:2, and 8:2 Cl-PFESA increased, while PFOS decreased
Shandong, China	[62]		PFOS, PFOA, PFHpA, PFNA, PFDA, PFUnDA, PFDoDA, PFTriDA, PFHxS	HFPO-TA, 4:2/6:2/8:2 Cl-PFESA	Detection rate and concentrations of alternative 4:2, 6:2, 8:2 Cl-PFESAs were higher than CHBM
Hubei, China	[43]	Dongfeng–Tongji cohort	PFOA, L-PFOS, PFHpA	PFBA	2008–2018: media concentrations: PFOA increased; L-PFOS decreased; detection rate: PFBA, PFHpA increased

**Table 2:** Concentration ranges of PFAS in human serum across selected biomonitoring studies: general-exposure and high-exposure groups.

		General Exposure Min–Max (ng/mL)	High Exposure Min–Max (ng/mL)
PFCA	TFA	<LOD-77	
	PFBA	<LOD-2.5	<LOD-23.9
	PFPrA	<LOD-6.1	
	PFPeA	<LOD-2.9	<LOD-3.882
	PFHxA	<LOD-2.7	<LOD-15.5
	PFHpA	<LOD-4.3	<LOD-884
	PFOA	<LOD-40.52	<LOD-32000
	PFNA	<LOD-5.8	<LOD-39.7
	PFDA	<LOD-6.9	<LOD-32.4
	PFUnA	<LOD-5.8	<LOD-21.8
	PFDoA	<LOD-0.5	<LOD-11.06
	PFTrA	<LOD-0.627	<LOD-0.8
	PFTeA	<LOD-0.21	<LOD-3.388
PFSA	PFBS	<LOD-2.6	<LOD-5967
	PFPeS	<LOD-0.034	<LOD-1873
	PFHxS	<LOD-16	<LOD-19837
	PFHpS	<LOD-0.73	<LOD-1113
	PFOS	<LOD-180	<LOD-62898
	PFNS	<LOD-0.0031	<LOD-754
	PFDS	<LOD-0.019	<LOD-19.3
PFESA	6:2 Cl-PFESA	<LOD-11.06	<LOD-173.1
	8:2 Cl-PFESA	<LOD-0.09	<LOD-4.6
FTSA	4:2 FTS		<LOD-3.96
	6:2 FTS		<LOD-152
	8:2 FTS		<LOD-0.22
	10:2 FTS		<LOD-0.11
FASA	FOSA	<LOD-0.36	<LOD-0.9
FASAA	MeFOSAA	<LOD-4.5	<LOD-0.42
	EtFOSAA	<LOD-6	<LOD-1.28

LOD = limit of detection. <LOD indicates that the reported concentration was below the analytical limit of detection in the original study. LOD values may vary across studies due to differences in instrumentation, sample preparation, and matrix effects.

**Table 3:** Concentration ranges of PFAS in human urine in general-exposure and high-exposure groups reported across selected biomonitoring studies.

		General Exposure Min–Max (ng/mL)	High Exposure Min–Max (ng/mL)
PFCA	TFA	<LOD-300	
	PFBA	<LOD-26	<LOD-449.90
	PFPeA	<LOD-34	<LOD-290.88
	PFHxA	<LOD-2.34	<LOD-189
	PFHpA	<LOD-11	<LOD-181
	PFOA	<LOD-21.5	<LOD-53.6
	PFNA	<LOD-7.118	<LOD-0.75
	PFDA	<LOD-30	<LOD-0.43
	PFUnA	<LOD-0.125	<LOD-0.13
	PFDoA	<LOD-0.166	<LOD-1.09
	PFTrA		<LOD-4.2
	PFTeA		<LOD-8.49
PFSA	PFBS	<LOD-2.13	<LOD-1800
	PFPeS	<LOD-0.022	<LOD-42.3
	PFHxS	<LOD-10.345	<OD-297.4
	PFHpS	<LOD-0.077	<LOD-1.97
	PFOS	<LOD-10.5	<LOD-81.5
	PFNS		<LOD-0.007
	PFDS	<LOD-0.34	<LOD-0.082
PFESA	6:2 Cl-PFESA	<LOD-0.000572	<LOD-0.03
FTSA	4:2 FTS		<LOD-5.42
	6:2 FTS		<LOD-21
	8:2 FTS		<LOD-0.007
	10:2 FTS		<LOD-0.017
FASA	FOSA		<LOD-2

LOD = limit of detection. <LOD indicates that the reported concentration was below the analytical limit of detection in the original study. LOD values may vary across studies due to differences in instrumentation, sample preparation, and matrix effects.

**Table 4:** Final dissociation constants (Kd, μM) of PFAS binding to HSA and hL-FABP used in the statistical analyses [35,82,168,170,175,176,177,178,179,180,181,182,183,184,185].

	Kd-HSA (μM)	Kd-FABP (μM)
PFBA	546.6	879
PFPeA	286.0	685
PFHxA	195.0	443.0
PFHpA	0.22	346.8
PFOA	9.7	18.3
PFNA	1.7	10.4
PFDA	1.3	31.5
PFUnA	1.4	23.9
PFDoA	103.0	12.3
PFTrA	3.64	317
PFTeA	14.0	60.5
PFBS	287.8	436.0
PFHxS	6.3	85.7
PFOS	32.8	11.5
6:2 FTSA	67.0	12.5
6:2 Cl-PFESA	1347.0	64.1
PFO3DA	546.6	879.0

Note: The Kd values listed represent the back-transformed medians of log10-transformed dissociation constants compiled from multiple sources of the literature. For studies reporting association constants (Ka), the corresponding Kd values were calculated as 1/Ka. All values were converted to µM prior to log10-transformation. The number of decimal places shown reflects the arithmetic output of the back-transformation and should not be interpreted as the precision of the underlying experimental measurements, which is governed by the inter-study variability of the original data.

**Table 5:** Pairwise Pearson correlation coefficients (r) between PFAS urine-to-blood concentration ratios (UtBCRs), protein-binding parameters, carbon chain length, and half-life.

	Pooled Populations UtBCR	High-Exposure UtBCR	General-Exposure UtBCR	Carbon Chain Length	Half-Life
BE-FABP	0.937 **	0.653 *	0.978 **	−0.968 **	−0.685 *
BE-HSA	0.853 **	0.565 *	0.934 **	−0.976 **	−0.566
BE-OAT1	0.888 **	0.578 *	0.962 **	−0.977 **	−0.657 *
BE-OAT3	0.928 **	0.636 *	0.960 **	−0.951 **	−0.608 *
BE-OAT4	0.902 **	0.631 *	0.940 **	−0.961 **	−0.65 *
BE-URAT1	0.909 **	0.679 **	0.973 **	−0.945 **	−0.643 *
Kd-HSA	0.571	0.367	0.670 *	−0.588 *	−0.636 *
Kd-FABP	0.804 **	0.807 **	0.802 **	−0.626 **	−0.72 **
Carbon chain length	−0.821 **	−0.527	−0.937 **	1	0.569
Half-life	−0.697 *	−0.733 *	−0.745 **	0.569	1

* *p* < 0.05; ** *p* < 0.01.

## Data Availability

No new data were created or analyzed in this study. Data sharing is not applicable to this article.

## Supplementary Materials

The following supporting information can be downloaded at https://www.mdpi.com/article/10.3390/molecules31111880/s1. Text S1: Molecular docking analysis; Text S2: Compilation and standardization of PFAS–protein dissociation constants; Table S1: List of Abbreviations; Table S2: Molecular formulas, chemical structural formulas, and number of carbon chain length for 16 substances; Table S3: Urine-to-blood concentration ratio (UtBCR) in high-exposure group; Table S4: Urine-to-blood concentration ratio (UtBCR) in general-exposure group; Table S5: Binding energies of PFAS–protein complexes predicted by molecular docking; Table S6: Literature-reported and converted dissociation constants (Kd, μM) for the binding of 16 PFAS to hL-FABP; Table S7: Literature-reported and converted dissociation constants (Kd, μM) for the binding of 16 PFAS to HSA; Table S8: Pairwise Pearson correlation coefficients (r) among UtBCR, protein binding parameters, carbon-chain length, and biological half-life of PFAS after exclusion of studies reporting only median concentrations; Figure S1: Performance of partial least squares regression (PLSR) models predicting UtBCR of PFAS in different exposure groups based on protein binding and transport-related variables, using the mean-only dataset.
