# Supplementary material for: Urine-to-Blood Partitioning of Per- and Polyfluoroalkyl Substances in Human Biomonitoring: Implications for Environmental Exposure Analysis and Bioaccumulation Assessment
Source: Molecules. 2026 May 30;31(11):1880. doi: 10.3390/molecules31111880 (PMC13257718; doi:10.3390/molecules31111880)
Supplement: Supplementary file 1 [file molecules-31-01880-s001.zip › molecules-4306461-supplementary.pdf]

Supporting Information

Urine-to-Blood Partitioning of Per- and Polyfluoroalkyl Substances in Human Biomonitoring: Implications for Environmental Exposure Analysis and Bioaccumulation Assessment

Peiyao Ye 1, Hexiang Bai 1, Jing Shi 2, Zhaomin Dong 1 and Kai Luo 1,*

1 Key Laboratory of Environmental Medical Engineering, Ministry of Education, School of Public Health, Southeast University, Nanjing 210009, China; 220234066@seu.edu.cn (P.Y.); 230268611@seu.edu.cn (H.B.); dongzm@seu.edu.cn (Z.D.)

2 Suzhou Center for Disease Control and Prevention, Suzhou 215000, China; lhjyk@szcdc.cn

* Correspondence: luokai@seu.edu.cn; Tel.: +86-25-83272561

Content of Supporting Information

Text S1. Molecular docking analysis.

Text S2. Compilation and standardization of PFAS–protein dissociation constants.

Table S1. List of Abbreviations

Table S2. Molecular formulas, chemical structural formulas, and number of carbon chain length for 16 substances.

Table S3. Urine-to-blood concentration ratio (UtBCR) in high-exposure group.

Table S4. Urine-to-blood concentration ratio (UtBCR) in general-exposure group.

Table S5. Binding energies of PFAS–protein complexes predicted by molecular docking.

Table S6. Literature-reported and converted dissociation constants (Kd, μM) for the binding of 16 PFAS to hL-FABP.

Table S7. Literature-reported and converted dissociation constants (Kd, μM) for the binding of 16 PFAS to HSA.

Table S8. Pairwise Pearson correlation coefficients (r) among UtBCR, protein binding parameters, carbon-chain length, and biological half-life of PFAS after exclusion of studies reporting only median concentrations.

Figure S1. Performance of partial least squares regression (PLSR) models predicting UtBCR of PFAS in different exposure groups based on protein

binding and transport-related variables, using the mean-only dataset.

Text S1. Molecular docking analysis

The 3D structures of 16 PFASs were retrieved from the PubChem database. Structural models of the target proteins, including human liver fatty acid-binding protein (hL-FABP; PDB ID: 3STM), human serum albumin (HSA; PDB ID: 6R7S), organic anion transporter 1 (OAT1; PDB ID: 9J04), organic anion transporter 4 (OAT4; PDB ID: 9M9Y), and urate transporter 1 (URAT1; PDB ID: 9DK9), were obtained from the Protein Data Bank. The three-dimensional structure of human OAT3 (UniProt ID: Q8TCC7) was obtained as an AlphaFold-predicted model from the AlphaFold Protein Structure Database (https://alphafold.ebi.ac.uk/), since no experimentally resolved structure of OAT3 is currently available. Prior to docking, protein structures were preprocessed in AutoDockTools 1.5.6 through removal of water molecules and addition of polar hydrogens. Molecular docking was performed in batch mode using AutoDock Vina, and binding affinities were recorded as binding energies (kcal/mol) to characterize the interaction strength between PFASs and the target proteins (**Table S4**). The predicted binding energies were expressed as negative values, and a larger absolute value corresponded to stronger binding and a lower tendency for dissociation.

Text S2. Compilation and standardization of PFAS–protein dissociation constants

Experimentally measured dissociation constants for PFAS binding to HSA and L-FABP, denoted as Kd-HSA and Kd-FABP, were compiled from the published literature. For studies that directly reported dissociation constants (Kd), the reported values were extracted as original data. For studies that provided association constants (Ka) instead of dissociation constants, the corresponding Kd values were calculated as the reciprocal of Ka according to Kd=1/Ka. All dissociation constants were converted to a uniform unit of μM prior to further analysis (**Table S5 and S6**).

To reduce inter-study variability and improve comparability among values obtained from different experimental systems and literature sources, all compiled Kd values were transformed using the base-10 logarithm. The $\log_{10}$-transformed values were then pooled for each PFAS–protein pair, and the median value was calculated to represent the central tendency of the compiled dataset. Finally, the median $\log_{10}$-transformed value was back-transformed by exponentiation to obtain the final Kd value used in the subsequent statistical analyses (**Table 3**).

Table S1. List of Abbreviations

Abbreviation	Full Name
PFAS	Per- and polyfluoroalkyl substances
PFCAs	Perfluorocarboxylic acid
PFSAs	Perfluorosulfonic Acid
PFAA	Perfluoroalkyl acids
UtBCR	Urine-to-blood concentration ratio
HSA	Human serum albumin
L-FABP	Liver fatty acid-binding protein
OAT	Organic anion transporter
URAT1	Urate Transporter 1
OECD	Organization for Economic Co-operation and Development
TFA	Trifluoroacetic acid
PFBA	Perfluorobutanoic acid
PFPrA	Pentafluoropropanoic acid
PFPeA	Perfluoropentanoic acid
PFHxA	Perfluorohexanoic acid
PFHpA	Perfluoroheptanoic acid
PFOA	Perfluorooctanoic acid
PFNA	Perfluorononanoic acid
PFDA	Perfluorodecanoic acid
PFUnDA	Perfluoroundecanoic acid
PFDoDA	Perfluorododecanoic acid
PFTriDA	Perfluorotridecanoic acid
PFTeA	Perfluorotetradecanoic acid
PFBS	Perfluorobutanesulfonic acid
PFPeS	Perfluoropentanesulfonic acid
PFHxS	Perfluorohexanesulfonic acid
PFHpS	Perfluoroheptanesulfonic acid
PFOS	Perfluorooctane sulfonate
PFNS	Perfluorononanesulfonic acid
PFDS	Perfluorodecane sulfonic acid
4:2 Cl-PFESA	4:2 Chlorinated Perfluoroalkyl Ether Sulfonate
6:2 Cl-PFESA	6:2 Chlorinated Perfluoroalkyl Ether Sulfonate
8:2 Cl-PFESA	8:2 Chlorinated Perfluoroalkyl Ether Sulfonate
4:2 FTS	4:2 Fluorotelomer sulfonic acid
6:2 FTS	6:2 Fluorotelomer sulfonic acid
8:2 FTS	8:2 Fluorotelomer sulfonic acid
10:2 FTS	10:2 Fluorotelomer sulfonic acid
FOSA	Perfluorooctanesulfonamide
MeFOSAA	Methyl perfluorooctane sulfonamido acetic acid
EtFOSAA	Ethyl perfluorooctane sulfonamido acetic acid
PFMOAA	Perfluoro-2-methoxyacetic acid

PFO2HxA	Perfluoro-3,5-dioxahexanoic acid
PFO3OA	Perfluoro(3,5,7-trioxaoctanoic) acid
HFPO-DA	Hexafluoropropylene oxide dimer acid
HFPO-TA	Hexafluoropropylene oxide trimer acid
ADONA	Dodecafluoro-3H-4,8-dioxanonanoate
cC6O4	2,2-Difluoro-2-((2,2,4,5-tetrafluoro-5-(trifluoromethoxy)-1,3-dioxolan-4-yl)oxy)acetic Acid Ammonium Salt
Kd	Dissociation constant
LOD	Limit of detection
MDL	Method detection limit
eGFR	Estimated glomerular filtration rate

Table S2. Molecular formulas, chemical structural formulas, and number of carbon chain length for 16 substances.

Analytes	Formula	Chemical structural	Carbon chain length
PFBA	$C_4HF_7O_2$	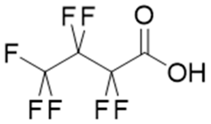	4
PFPeA	$C_5HF_9O_2$	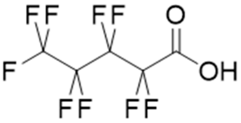	5
PFHxA	$C_6HF_{11}O_2$	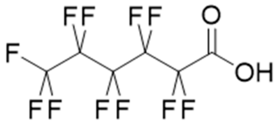	6
PFHpA	$C_7HF_{13}O_2$	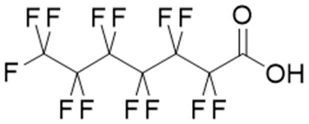	7
PFOA	$C_8HF_{15}O_2$		8
PFNA	$C_9HF_{17}O_2$	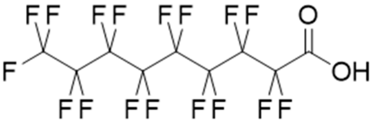	9

PFDA	$C_{10}HF_{19}O_2$	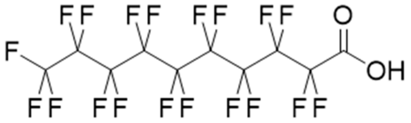	10
PFUdA	$C_{11}HF_{21}O_2$	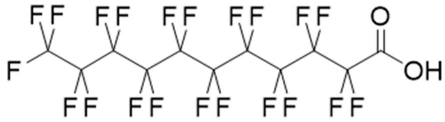	11
PFDoA	$C_{12}HF_{23}O_2$	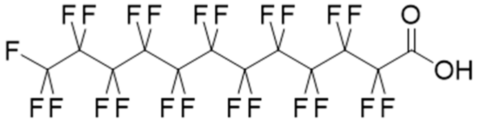	12
PFTrDA	$C_{13}HF_{25}O_2$	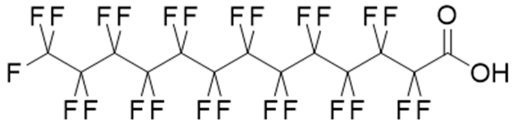	13
PFTeDA	$C_{14}HF_{27}O_2$	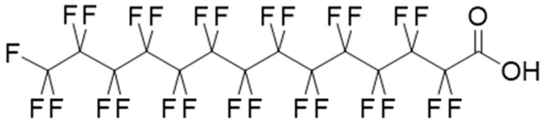	14
PFBS	$C_4HF_9O_3S$	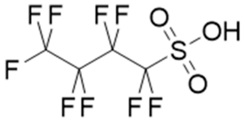	4
PFHxS	$C_6HF_{13}O_3S$	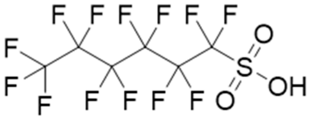	6
PFOS	$C_8HF_{17}O_3S$	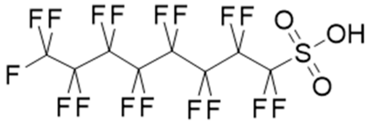	8
6:2 Cl-PFESA	$C_8HClF_{16}O_4S$	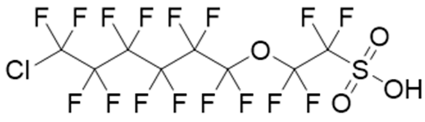	8
HFPO-DA	$C_6HF_{11}O_3$	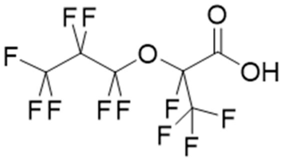	6

Table S3. Urine-to-blood concentration ratio (UtBCR) in high-exposure group.

	Peng et al. 2022[122]	Worley et al. 2017[45]	He et al. 2023[102]	Wang et al. 2018[128]	Xu et al. 2020[41]	Gao et al. 2015[130]	Liu et al. 2021[139]	Lei et al. 2023[140]	Fu et al. 2016[134]	Zhou et al. 2014[129]		Li et al. 2022[43]		
										Fishery employee	Fishery family	Round 5	Round6	Round10
Geographical Location	Guang zhou	Alabama	Hubei	Wuhan	Arvidsjaur	Hubei	Guangzhou	Shandong	Hubei	Hubei		Ronneby		
Sample Size	163	45	72	8	17	36	169	60	302	39	7	62		
Age (year)	20-63	Average=52	-	-	24-62	-	-	-	19-65	33-58	36-63	-		
Exposure Types	Waste Recycling Plants Workers	Residentially Exposed	Fluorochemical Manufactory Workers	Fishery Employees	Airport Employees	Fluorochemical Plant Workers	Waste Recycling Workers	Petrochemical Worker	Fluorochemical Workers	Fishery Employee	Fishery Family	High Exposure to Drinking Water		
PFBA	10.64402		3.21429	0.39612			10.65990	0.28333		0.24188	0.21139			
PFPeA	14.88277						14.85915	1.76083						
PFHxA	0.04930							0.15421		0.12195				
PFHpA	1.62208		0.33276	0				0.00519		0.14786	0.27250			
PFOA	0.51927	0.00231	0.00495	0.00413	0.00310	0.00315		0.00169	0.00409	0.00308	0.00305	0.00208	0.0025	0.00254
PFNA	0	0.00875	0.00230	0				0.00230		0.00297	0.00645			
PFDA	0.11626		0.00040	0				0.00326		0.00071	0.00230			
PFUdA	0		0.00056	0				0		0	0			
PFDoA	0.18177		0.00113	0				0.00606		0	0			
PFTrDA									0.00555					
PFTeDA									0.09785					
PFBS	2.46814		0.40602	0.31754				0.00511		0.18394	0.14744			
PFHxS	0.06533	0.01831	0.00317	0.00069	0.00078	0.00327		0.00174	0.00210	0.00076	0.00035	0.00082	0.00081	0.00093
PFOS	0.03739	0.00603	0.30936	0.00087		0.00048	0.04244	0.00150	0.00078	0.00070	0.00079			
6:2 Cl-PFESA									0.00118					
HFPO-DA														

Table S4. Urine-to-blood concentration ratio (UtBCR) in general-exposure group.

	Zheng et al. 2023[46]	Kim et al. 2019[125]	Wang et al. 2018[126]	Zhang et al. 2013[40]	Kim et al. 2014[124]	Li et al. 2013[127]	Zhou et al. 2014[129]	Gu et al. 2019[138]	Yao et al. 2023[133]	Chen et al. 2022[123]	Zhang et al. 2015[86]		Harada et al. 2005[132]				NHANES 2013-2014	
											Adult	Maternal	Young male	Young femal	Old male	Old femal	3-11 years	12+ years old
Geographical Location	Indiana	Seoul and Busan	Shenzhen	Shijiazhuang and Handan	Dae-gu	Shanxi	Hubei	Shandong	Huantai and Hangzhou	Tianjin	Tianjin		Kyoto				United States	
Sample Size	81	94	39	86	120	64	9	292	80	20	54	27	5	5	5	5	112	1921
Age (year)	49±16	2-82	20-53	21-85	5-13	19-53	26-56	Meidan=30.5	infants	20-25	22-62	21-39	Mean=21.6	23	67.8	68.8	3-11	12+
PFBA	1.7368				0													
PFPeA	6400	4.5833			4.7083				0.9185									
PFHxA	0.0294	1.1539			2.0708					0.0175								
PFHpA	0.0313	0.4		0.0224	4.3269			3.6696	0.0181	0.0201								
PFOA	0.2381	0.0361	0.0020	0.0261	0	0.0057	0.0055	0.0286	0.002	0.0086	0.0023	0.001	2.0506	1.114	1.8167	1.4143		
PFNA	0.0024	0.081	0.0006	0.0049	0		0	1.359	0.0015	0.0082							0.0751	0.0787
PFDA	0	0.2564	0.0006	0.0024	0		0	0.0725	0.0008	4.4286								
PFUdA	0		0.0011		0		0	0.0651	0								0.6369	0.29
PFDoA	0				0		0	0.2979	0									
PFTrDA	0				0													
PFTeDA	0				0													
PFBS	0.01	2.1404			4.6857		0.2367	3.468	0.0493								0.9561	0.9905
PFHxS	0	0.071	0.0099	0.0009	0		0.035	7.0871		0.0019							0.0594	0.0354
PFOS	0.0003	0	0.0019	0.0012	0	0.0033	0.0008	0.2151	0	0.0038	0.001	0.0006	0.5635	0.9375	0.5361	0.9106		
6:2 Cl-PFESA									0.0003	0.0016								
HFPO-DA									0.0636									

Table S5. Binding energies of PFAS–protein complexes predicted by molecular docking.

	BE-HSA	BE-hL-FABP	BE-OAT1	BE-OAT3	BE-OAT4	BE-URAT1
PFBA	-6.212	-6.593	-5.398	-6.099	-5.697	-6.49
PFPeA	-6.987	-7.143	-5.885	-6.955	-5.953	-6.894
PFHxA	-7.502	-7.713	-6.433	-7.359	-6.654	-7.879
PFHpA	-7.828	-8.66	-6.903	-7.621	-6.791	-8.257
PFOA	-7.624	-8.989	-7.118	-7.56	-7.233	-8.695
PFNA	-8.644	-9.514	-7.933	-8.641	-7.726	-8.98
PFDA	-8.687	-10.367	-8.171	-8.955	-7.804	-9.772
PFUdA	-9.552	-10.753	-8.673	-9.295	-8.008	-9.994
PFDoA	-9.62	-10.776	-8.663	-9.584	-8.299	-10.764
PFTrDA	-9.757	-10.567	-9.227	-9.762	-8.589	-10.317
PFTeDA	-9.928	-10.858	-8.861	-9.679	-8.75	-9.373
PFBS	-6.579	-7.565	-6.087	-6.948	-5.766	-7.173
PFHxS	-7.65	-8.926	-6.866	-7.972	-7.317	-7.998
PFOS	-8.326	-9.423	-8.063	-8.72	-7.976	-9.078
6:2 Cl-PFESA	-8.515	-10.043	-7.745	-8.955	-7.554	-9.136
HFPO-DA	-7.271	-7.769	-6.453	-7.487	-6.609	-7.295

Table S6. Literature-reported and converted dissociation constants (Kd, μM) for the binding of 16 PFAS to hL-FABP.

	Khazaee et al. 2021[177]	Zhang et al. 2013[170]	Jia et al. 2022[36]	Gao et al. 2019[83]	Sheng et al. 2018[172]	Yang et al. 2020[178]
PFBA				879		
PFPeA				685		
PFHxA			538.07	443		140.76
PFHpA		336		358		
PFOA	0.099	50.4	17.95	197	8.03	18.74
PFNA		16.2	6.62	90		4.3
PFDA		12.9		77		
PFUdA		10.6		54		
PFDoA		12.3	3.45	95		
PFTrDA				317		
PFTeDA		60.5				
PFBS		1034		436		116.4
PFHxS	1.7	85.7		210		
PFOS	0.18	18.5	16.45	81	4.99	8.1
6:2 Cl-PFESA			12.47	102	4.05	
HFPO-DA			267.78		15.36	

Table S7. Literature-reported and converted dissociation constants (Kd, µM) for the binding of 16 PFAS to HSA.

	Starnes et al. 2024[179]	Beesoon et al. 2015[180]	Peng et al. 2024[181]	Jackson et al. 2021[182]	Jia et al. 2022[36]	Hebert et al. 2010[183]	Gao et al. 2019[184]	Li et al. 2021[185]	Gao et al. 2019[83]	Sheng et al. 2018[186]	Wu et al. 2024[187]
PFBA	2640			2570			713	0.079	419		49.092
PFPeA				2100				0.079	286		
PFHxA	1710			1640	195			0.025	195		
PFHpA			0.221	440		0.108		0.003	172		
PFOA	790.0	0.8	0.004	830	115	0.045		0.010	115		9.676
PFNA			0.000	580	73	0.039		0.004	73		
PFDA				1190		0.024		0.013	65		
PFUdA				1360		0.024		0.025	79		
PFDoA				1890	103			0.032	103		
PFTrDA								0.050	265		
PFTeDA							196	1.000			
PFBS	1680			1650			490	0.025	169		8.584
PFHxS				710		0.081		0.010	79		6.332
6:2 Cl-PFESA					67				67	16.7	
HFPO-DA	1570			1600	1156						16.952

Table S8. Pairwise Pearson correlation coefficients (r) among UtBCR, protein binding parameters, carbon-chain length, and biological half-life of PFAS after exclusion of studies reporting only median concentrations.

	Pooled populations UtBCR	High-exposure UtBCR	General-exposure UtBCR	Carbon chain length	Half-life
BE-FABP	0.754**	0.653*	0.934**	-0.968**	-0.685*
BE-HSA	0.688**	0.565*	0.846**	-0.976**	-0.566
BE-OAT1	0.727**	0.578*	0.890**	-0.977**	-0.657*
BE-OAT3	0.781**	0.636*	0.913**	-0.951**	-0.608*
BE-OAT4	0.754**	0.631*	0.857**	-0.961**	-0.65*
BE-URAT1	0.771**	0.679**	0.907**	-0.945**	-0.643*
Kd-HSA	0.451	0.367	0.516	-0.588*	-0.636*
Kd-FABP	0.789**	0.807**	0.835**	-0.626**	-0.72**
Carbon chain length	-0.653*	-0.527	-0.848**	1	0.569
Half-life	-0.697*	-0.733*	-0.764**	0.569	1

* $p < 0.05$; ** $p < 0.01$.

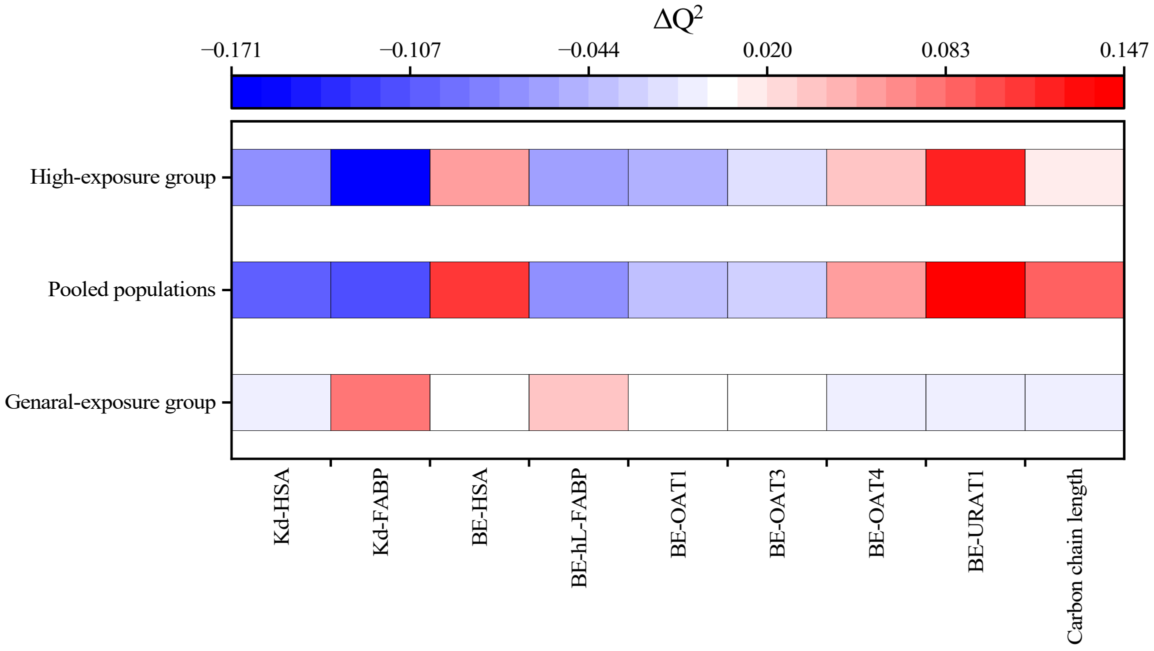

Figure S1. Performance of partial least squares regression (PLSR) models predicting UtBCR of PFAS in different exposure groups based

on protein binding and transport-related variables, using the mean-only dataset.

References:

36. Jia, Y.; Zhu, Y.; Xu, D.; Feng, X.; Yu, X.; Shan, G.; Zhu, L. Insights into the Competitive Mechanisms of Per- and Polyfluoroalkyl Substances Partition in Liver and Blood. Environ Sci Technol 2022, 56, 6192–6200, doi:10.1021/acs.est.1c08493.

40. Zhang, Y.; Beesoon, S.; Zhu, L.; Martin, J.W. Biomonitoring of Perfluoroalkyl Acids in Human Urine and Estimates of Biological Half-Life. Environ Sci Technol 2013, 47, 10619–10627, doi:10.1021/es401905e.

41. Xu, Y.; Fletcher, T.; Pineda, D.; Lindh, C.H.; Nilsson, C.; Glynn, A.; Vogs, C.; Norstrom, K.; Lilja, K.; Jakobsson, K.; et al. Serum Half-Lives for Short- and Long-Chain Perfluoroalkyl Acids after Ceasing Exposure from Drinking Water Contaminated by Firefighting Foam. Environ Health Perspect 2020, 128, 77004, doi:10.1289/EHP6785.

43. Li, Y.; Andersson, A.; Xu, Y.; Pineda, D.; Nilsson, C.A.; Lindh, C.H.; Jakobsson, K.; Fletcher, T. Determinants of serum half-lives for linear and branched perfluoroalkyl substances after long-term high exposure—A study in Ronneby, Sweden. Environ Int 2022, 163, 107198, doi:https://doi.org/10.1016/j.envint.2022.107198.

45. Worley, R.R.; Moore, S.M.; Tierney, B.C.; Ye, X.; Calafat, A.M.; Campbell, S.; Woudneh, M.B.; Fisher, J. Per- and polyfluoroalkyl substances in human serum and urine samples from a residentially exposed community. Environ Int 2017, 106, 135–143, doi:10.1016/j.envint.2017.06.007.

46. Zheng, G.; Eick, S.M.; Salamova, A. Elevated Levels of Ultrashort- and Short-Chain Perfluoroalkyl Acids in US Homes and People. Environ Sci Technol 2023, 57, 15782–15793, doi:10.1021/acs.est.2c06715.

83. Gao, K.; Zhuang, T.; Liu, X.; Fu, J.; Zhang, J.; Fu, J.; Wang, L.; Zhang, A.; Liang, Y.; Song, M.; et al. Prenatal Exposure to Per- and Polyfluoroalkyl Substances (PFASs) and Association between the Placental Transfer Efficiencies and Dissociation Constant of Serum Proteins-PFAS Complexes. Environ Sci Technol 2019, 53, 6529–6538, doi:10.1021/acs.est.9b00715.

86. Zhang, T.; Sun, H.; Qin, X.; Gan, Z.; Kannan, K. PFOS and PFOA in paired urine and blood from general adults and pregnant women: assessment of urinary elimination. Environ Sci Pollut Res Int 2015, 22, 5572–5579, doi:10.1007/s11356-014-3725-7.

102. He, A.; Li, J.; Li, Z.; Lu, Y.; Liang, Y.; Zhou, Z.; Man, Z.; Lv, J.; Wang, Y.; Jiang, G. Novel Insights into the Adverse Health Effects of per- and Polyfluoroalkyl Substances on the Kidney via Human Urine Metabolomics. Environ Sci Technol 2023, 57, 16244–16254, doi:10.1021/acs.est.3c06480.

122. Peng, L.; Xu, W.; Zeng, Q.; Sun, F.; Guo, Y.; Zhong, S.; Wang, F.; Chen, D. Exposure to perfluoroalkyl substances in waste recycling workers: Distributions in paired human serum and urine. Environ Int 2022, 158, 106963, doi:10.1016/j.envint.2021.106963.

123. Chen, X.; Feng, X.; Sun, X.; Li, Y.; Yang, Y.; Shan, G.; Zhu, L. Quantifying Indirect Contribution from Precursors to Human Body Burden of Legacy PFASs Based on Paired Blood and One-Week Duplicate Diet. Environ Sci Technol 2022, 56, 5632–5640, doi:10.1021/acs.est.1c07465.

124. Kim, D.H.; Lee, M.Y.; Oh, J.E. Perfluorinated compounds in serum and urine samples from children aged 5-13 years in South Korea. Environ Pollut 2014, 192, 171–178, doi:10.1016/j.envpol.2014.05.024.

125. Kim, D.H.; Lee, J.H.; Oh, J.E. Perfluoroalkyl acids in paired serum, urine, and hair samples: Correlations with demographic factors and dietary habits. Environ Pollut 2019, 248, 175–182, doi:10.1016/j.envpol.2019.02.017.

126. Wang, Y.; Zhong, Y.; Li, J.; Zhang, J.; Lyu, B.; Zhao, Y.; Wu, Y. Occurrence of perfluoroalkyl substances in matched human serum, urine, hair and nail. J Environ Sci (China) 2018, 67, 191–197, doi:10.1016/j.jes.2017.08.017.

127. Li, J.; Guo, F.; Wang, Y.; Zhang, J.; Zhong, Y.; Zhao, Y.; Wu, Y. Can nail, hair and urine be used for biomonitoring of human exposure to perfluorooctane sulfonate and perfluorooctanoic acid? Environ Int 2013, 53, 47–52, doi:10.1016/j.envint.2012.12.002.

128. Wang, Y.; Shi, Y.; Vestergren, R.; Zhou, Z.; Liang, Y.; Cai, Y. Using hair, nail and urine samples for human exposure assessment of legacy and emerging per- and polyfluoroalkyl substances. Sci Total Environ 2018, 636, 383–391, doi:10.1016/j.scitotenv.2018.04.279.

129. Zhou, Z.; Shi, Y.; Vestergren, R.; Wang, T.; Liang, Y.; Cai, Y. Highly elevated serum concentrations of perfluoroalkyl substances in fishery employees from Tangxun lake, china. Environ Sci Technol 2014, 48, 3864–3874, doi:10.1021/es4057467.

130. Gao, Y.; Fu, J.; Cao, H.; Wang, Y.; Zhang, A.; Liang, Y.; Wang, T.; Zhao, C.; Jiang, G. Differential accumulation and elimination behavior of perfluoroalkyl Acid isomers in occupational workers in a manufactory in China. Environ Sci Technol 2015, 49, 6953–6962, doi:10.1021/acs.est.5b00778.

132. Harada, K.; Inoue, K.; Morikawa, A.; Yoshinaga, T.; Saito, N.; Koizumi, A. Renal clearance of perfluorooctane sulfonate and perfluorooctanoate in humans and their species-specific excretion. Environ Res 2005, 99, 253–261, doi:10.1016/j.envres.2004.12.003.

133. Yao, J.; Dong, Z.; Jiang, L.; Pan, Y.; Zhao, M.; Bai, X.; Dai, J. Emerging and Legacy Perfluoroalkyl Substances in Breastfed Chinese Infants: Renal Clearance, Body Burden, and Implications. Environ Health Perspect 2023, 131, 37003, doi:10.1289/EHP11403.

134. Fu, J.; Gao, Y.; Cui, L.; Wang, T.; Liang, Y.; Qu, G.; Yuan, B.; Wang, Y.; Zhang, A.; Jiang, G. Occurrence, temporal trends, and half-lives of perfluoroalkyl acids (PFAAs) in occupational workers in China. Sci Rep 2016, 6, 38039, doi:10.1038/srep38039.

138. Gu, J. The investigations of human exposure characteristic and health risk of typical merging contaminants. Shanghai Jiao Tong University, Shanghai, China, 2018.

139. Liu, S. Distribution characteristics of per- and polyfluoroalkyl substances (PFASs) in leachate, fly ash and bottom ash in waste incineration plants and exposure analysis of PFASs to waste recycling workers. Jinan University, Guangzhou, China, 2021.

140. Tao, L. Pollution characteristics and human exposure of per- and polyfluoroalkyl substances (PFAS) in petrochemical parks. Chinese Research Academy of Environmental Sciences, Beijing, China, 2023.

170. Zhang, L.; Ren, X.M.; Guo, L.H. Structure-based investigation on the interaction of perfluorinated compounds with human liver fatty acid binding protein. Environ Sci Technol 2013, 47, 11293–11301, doi:10.1021/es4026722.

172. Sheng, N.; Cui, R.; Wang, J.; Guo, Y.; Wang, J.; Dai, J. Cytotoxicity of novel fluorinated alternatives to long-chain perfluoroalkyl substances to human liver cell line and their binding capacity to human liver fatty acid binding protein. Arch Toxicol 2018, 92, 359–369, doi:10.1007/s00204-017-2055-1.

177. Khazaee, M.; Christie, E.; Cheng, W.; Michalsen, M.; Field, J.; Ng, C. Perfluoroalkyl Acid Binding with Peroxisome Proliferator-Activated Receptors α, γ, and δ, and Fatty Acid Binding Proteins

by Equilibrium Dialysis with a Comparison of Methods. Toxics 2021, 9, doi:10.3390/toxics9030045.
178. Yang, D.; Han, J.; Hall, D.R.; Sun, J.; Fu, J.; Kutarna, S.; Houck, K.A.; LaLone, C.A.; Doering, J.A.; Ng, C.A.; et al. Nontarget Screening of Per- and Polyfluoroalkyl Substances Binding to Human Liver Fatty Acid Binding Protein. Environ Sci Technol 2020, 54, 5676–5686, doi:10.1021/acs.est.0c00049.
179. Starnes, H.M.; Jackson, T.W.; Rock, K.D.; Belcher, S.M. Quantitative cross-species comparison of serum albumin binding of per- and polyfluoroalkyl substances from five structural classes. Toxicol Sci 2024, 199, 132–149, doi:10.1093/toxsci/kfae028.
180. Beesoon, S.; Martin, J.W. Isomer-Specific Binding Affinity of Perfluorooctanesulfonate (PFOS) and Perfluorooctanoate (PFOA) to Serum Proteins. Environ Sci Technol 2015, 49, 5722–5731, doi:10.1021/es505399w.
181. Peng, M.; Xu, Y.; Wu, Y.; Cai, X.; Zhang, W.; Zheng, L.; Du, E.; Fu, J. Binding Affinity and Mechanism of Six PFAS with Human Serum Albumin: Insights from Multi-Spectroscopy, DFT and Molecular Dynamics Approaches. Toxics 2024, 12, doi:10.3390/toxics12010043.
182. Jackson, T.W.; Scheibly, C.M.; Polera, M.E.; Belcher, S.M. Rapid Characterization of Human Serum Albumin Binding for Per- and Polyfluoroalkyl Substances Using Differential Scanning Fluorimetry. Environ Sci Technol 2021, 55, 12291–12301, doi:10.1021/acs.est.1c01200.
183. Hebert, P.C.; MacManus-Spencer, L.A. Development of a fluorescence model for the binding of medium- to long-chain perfluoroalkyl acids to human serum albumin through a mechanistic evaluation of spectroscopic evidence. Anal Chem 2010, 82, 6463–6471, doi:10.1021/ac100721e.
184. Gao, K.; Fu, J.; Xue, Q.; Fu, J.; Fu, K.; Zhang, A.; Jiang, G. Direct determination of free state low molecular weight compounds in serum by online TurboFlow SPE HPLC-MS/MS and its application. Talanta 2019, 194, 960–968, doi:10.1016/j.talanta.2018.10.082.
185. Li, W.; Hu, Y.; Bischel, H.N. In-Vitro and In-Silico Assessment of Per- and Polyfluoroalkyl Substances (PFAS) in Aqueous Film-Forming Foam (AFFF) Binding to Human Serum Albumin. Toxics 2021, 9, doi:10.3390/toxics9030063.
186. Sheng, N.; Wang, J.; Guo, Y.; Wang, J.; Dai, J. Interactions of Perfluorooctanesulfonate and 6:2 Chlorinated Polyfluorinated Ether Sulfonate with Human Serum Albumin: A Comparative Study. Chem Res Toxicol 2020, 33, 1478–1486, doi:10.1021/acs.chemrestox.0c00075.
187. Wu, Y.; Bao, J.; Liu, Y.; Wang, X.; Lu, X.; Wang, K. In Vitro and In Silico Analysis of the Bindings between Legacy and Novel Per- and Polyfluoroalkyl Substances and Human Serum Albumin. Toxics 2024, 12, doi:10.3390/toxics12010046.
